# A nirK–cbb3 genomic region links SAR11 to nitrogen loss in the northern Benguela Upwelling System

**DOI:** 10.1099/mgen.0.001620

**Published:** 2026-01-19

**Authors:** Robert M. Morris, Timothy E. Mattes, Kunmanee Bubphamanee, Mike C. Sadler, Luke A. Calderaro, Jen Karolewski, Karen L. Casciotti, Olivia U. Mason

**Affiliations:** 1 School of Oceanography, University of Washington, Seattle, WA, USA; 2 Department of Civil and Environmental Engineering, 4105 Seamans Center, University of Iowa, Iowa City, IA 52242, USA; 3 Department of Earth and Space Sciences, University of Washington, Seattle, WA, USA; 4 Oceans Department, Stanford University, Stanford, CA 94305, USA; 5 Department of Earth, Ocean and Atmospheric Science, Florida State University, Tallahassee, FL, USA

**Keywords:** bacteria, denitrification, marine, northern Benguela Upwelling System (nBUS), oxygen minimum zone (OMZ), SAR11

## Abstract

Denitrification leads to nitrogen loss from marine oxygen minimum zones. The complete metabolic pathway for denitrification reduces nitrate to dinitrogen gas in four sequential steps. Many facultatively anaerobic bacteria are capable of the initial step of nitrate reduction to nitrite. Far fewer contribute to nitrogen loss by reducing nitrite to nitric oxide, nitrous oxide or dinitrogen gas. We sequenced the genomes of 24 bacteria isolated from low-oxygen waters (23 µM) in the northern Benguela Upwelling System (nBUS) to identify species with the genetic potential for denitrification. Most isolates have the genetic potential for partial denitrification, including ten SAR11 strains with a genomic region that codes for a copper-containing nitrite reductase (NirK) and a high-affinity cbb3-type cytochrome c oxidase. Evidence that nBUS SAR11 have the potential to respire nitrite in low-dissolved-oxygen waters suggests that they could have a more direct role in marine nitrogen loss.

**Impact Statement**

Genomic diversity has hindered efforts to identify the genetic potential for denitrification among bacteria in marine oxygen minimum zones. We used a cultivation-based, high-throughput genomics approach to overcome this limitation and identify bacteria with the genetic potential to contribute to nitrogen loss. Most of the bacteria we sequenced (79%) have the potential to contribute to denitrification, including all SAR11 strains.

## Data Summary

Genome sequences were deposited in GenBank under BioProject PRJNA1173757, with accession and sequence read archive identifiers for each genome (Table S1).

## Introduction

In marine oxygen minimum zones (OMZs), the concentrations of dissolved oxygen (DO) can fall to less than 10 nmol l^−1^ [1]. Although OMZs account for only 1% of the ocean by volume, microbial processes occurring there catalyse 30–50% of marine nitrogen loss [2,3]. This outsized impact on the marine nitrogen budget is due largely to the anaerobic microbial processes of denitrification and anaerobic ammonia oxidation (anammox) that occur in these low-oxygen waters [4,6].

Denitrification is the stepwise reduction of nitrate (NO_3_^−^) to nitrite (NO_2_^−^), nitric oxide (NO), nitrous oxide (N_2_O) and di-nitrogen gas (N_2_) [7]. Each step in the denitrification pathway requires a specific enzyme, and it is common for microbes to possess some, but not all, of the required enzymes for denitrification [8]. While many anaerobic or facultatively anaerobic bacteria can respire nitrate, fewer have the genetic potential to reduce nitrite to a gas (NO, N_2_O or N_2_). This incomplete denitrification can lead to the accumulation of intermediates in anoxic waters and fuel competition between microbes specializing in components of the denitrification pathway [9]. Low-oxygen waters with these microbes can be either a source or a sink of N_2_O, a potent greenhouse gas, depending on environmental conditions and interactions among community members.

The Benguela Upwelling System (BUS) is an important and relatively understudied OMZ in the South Atlantic Ocean. Several studies have documented significant production of nitrous oxide within the low-oxygen waters of the BUS [10,13]. While many heterotrophic bacteria respire nitrogen oxides, there are only a few cultures or complete genome sequences from the BUS, and the micro-organisms responsible for this nitrogen loss are still largely unknown [13]. SAR11, one of the most abundant lineages of bacteria in the oceans [14], is also found in these low-oxygen waters, but studies of SAR11 and facultatively anaerobic bacteria in the BUS are lacking. While nitrate reduction by SAR11 has not yet been demonstrated in culture or *in situ,* there is evidence that they express genes for nitrate respiration in the Eastern tropical North Pacific (ETNP) OMZ [15]. This suggests that SAR11 could contribute to nitrogen loss in the ocean through the production of nitrite, a key substrate for denitrification and anammox. This capacity would extend their known ecological role and increase their importance in anoxic waters of marine OMZs. We cultured and sequenced the complete genomes of 24 bacteria from low-dissolved-oxygen waters (23 µM) in the northern BUS (nBUS) OMZ to identify the potential for SAR11 and other bacteria to contribute to denitrification.

## Methods

### High-throughput cultivation

We used a high-throughput dilution-to-extinction approach to isolate bacteria from a low-dissolved-oxygen (24 µmol kg^−1^) sample collected on 15 April 2024, at a depth of 225 m in the BUS (22°59'58.2"S 13°30'E), as previously described [16]. Briefly, 1 ml of seawater was amended with filter-sterilized glycerol to a final concentration of 10% (v/v), flash-frozen in liquid nitrogen and stored at −80 °C. The sample was later thawed on ice, and cells were diluted to 5 and 50 cells per millilitre in 1 l of 30 kDa filter-sterilized Puget Sound seawater amended with 100 µM of thiosulphate to provide an energy source for SUP05 and other autotrophic bacteria with the potential to respire nitrogen oxides. Two millilitres of the diluted cell suspension was then added to each well of a 96-well Teflon plate (three plates per dilution, totalling 576 wells) and incubated at 13 °C in the dark for 4 weeks. Sterile media was used to determine the limit of detection for cell counts, eliminating the need for uninoculated control wells. Wells were screened for growth each week using a Guava easyCyte flow cytometer (Cytek, Fremont, CA, USA). Bacteria in wells that were positive for growth (>10^5^ cells per millilitre) were transferred to 1 l acid-washed and autoclaved polycarbonate bottles containing filter-sterilized seawater amended with 100 µm thiosulphate and monitored for growth until they reached between 10^5^ and 10^6^ cells per millilitre. Cells were then filtered onto 47 mm 0.2 µm isopore membrane filters (MilliporeSigma, Burlington, MA, USA) and stored at −80 °C. A total of 51 cultures met our criteria for sequencing. This included representatives from cultures that reached similar cell densities and had similar flow cytometry profiles after 2 weeks, as well as isolates from all culture wells that were positive for growth after 3 weeks, and that produced >50 ng of DNA following extraction. No additional prescreening was conducted.

### High molecular weight DNA extraction

Filters with cells from cultures were placed in sterile 2 ml DNA LoBind tubes (Eppendorf, Hamburg, Germany) and cut into smaller pieces with sterile scissors. Sterile Tris-EDTA (TE) buffer (200 µl) was added, and filters were frozen at −80 °C for 20 min and then thawed at room temperature. High molecular weight DNA was extracted from the filters using the Autogen QuickGene DNA Tissue Kit (Autogen, Holliston, MA, USA), modified by doubling all recommended extraction volumes and eluting DNA in 200 µl of molecular grade water. DNA was purified using 1× v/v DNA magnetic beads (Sergi Lab Supplies, Seattle, WA, USA) with two 80% ethanol washes, then eluted in 20 µl of molecular grade water. Purified DNA was quantified using a Qubit dsDNA Quantitation High Sensitivity kit (Invitrogen, Waltham, MA, USA).

### Long-read genome sequencing and assembly

High molecular weight DNA libraries for whole-genome sequencing were prepared using the SQK-RAD114 rapid library prep kit, following the recommended protocol (Oxford Nanopore Technologies, Oxford, UK), and sequenced on a MinION equipped with Flongle R10.4.1 flow cells (Oxford Nanopore Technologies, Oxford, UK). No additional size-selection step was included. DNA bases were called using the Dorado basecalling algorithm (dna_r10.4.1_e8.2_400bps_hac@v4.3.0), which removed reads with quality scores <Q9, reads with <200 bp and adaptors. Only genomes that produced a circular contig from fastq files assembled using Flye v2.9.1 [17] were subsequently polished with Medaka v1.7.2 (github.com/nanoporetech/medaka) [18]. For submission to National Center for Biotechnology Information (NCBI) and analytical purposes, single circular contigs produced by Flye and polished with Medaka were reoriented to create linear contigs starting with the *dnaA* gene, using DFAST (dfast.ddbj.nig.ac.jp) [19].

### Genome identification

Annotation was completed by NCBI using the Prokaryotic Genome Annotation Pipeline v6.8 [20,21]. Bacterial taxonomy was determined using DFAST (dfast.ddbj.nig.ac.jp) [19] and NCBI annotations. All isolates were given the prefix ‘nBUS’ followed by the cultivation number. SAR11 species were identified by average nucleotide identity (ANI) with Pan-genome Explorer [22,23]. Phylogenetic trees were constructed using CLUSTALW amino acid alignments and RAxML v8.2.11 with model GTRGAMMA [24]. A whole-genome phylogeny was constructed using the bacterial_71 single-copy core gene collection in anvi’o v8 [25].

### Denitrification potential

Genomes were re-annotated for comparative analysis of denitrification genes using DRAM (ver. 1.1.1) [26] with the KEGG [27], UniRef90 [28] and PFAM [29] databases. Metagenome-assembled genomes (MAGs) related to SAR11 and with *nirK* genes were identified using blast. Briefly, NirK from SAR11 nBUS_025 was used to search the NCBI non-redundant protein database with a cutoff score of 1e-150 and a percent similarity score of >90. A MAG from Saanich Inlet with a percent identity score of 74% was also identified and included in subsequent analyses. Related NirK proteins from well-characterized denitrifying organisms were identified by searching the AlphaFold protein structure database with SAR11 nBUS_025 NirK. AlphaFold hits were restricted to the highest quality matches with average pLDDT metric confidence scores >90 in UniProt reference proteomes [30,31].

## Results and discussion

### Denitrification potential of bacteria isolated from the nBUS

**Fig. 1.**
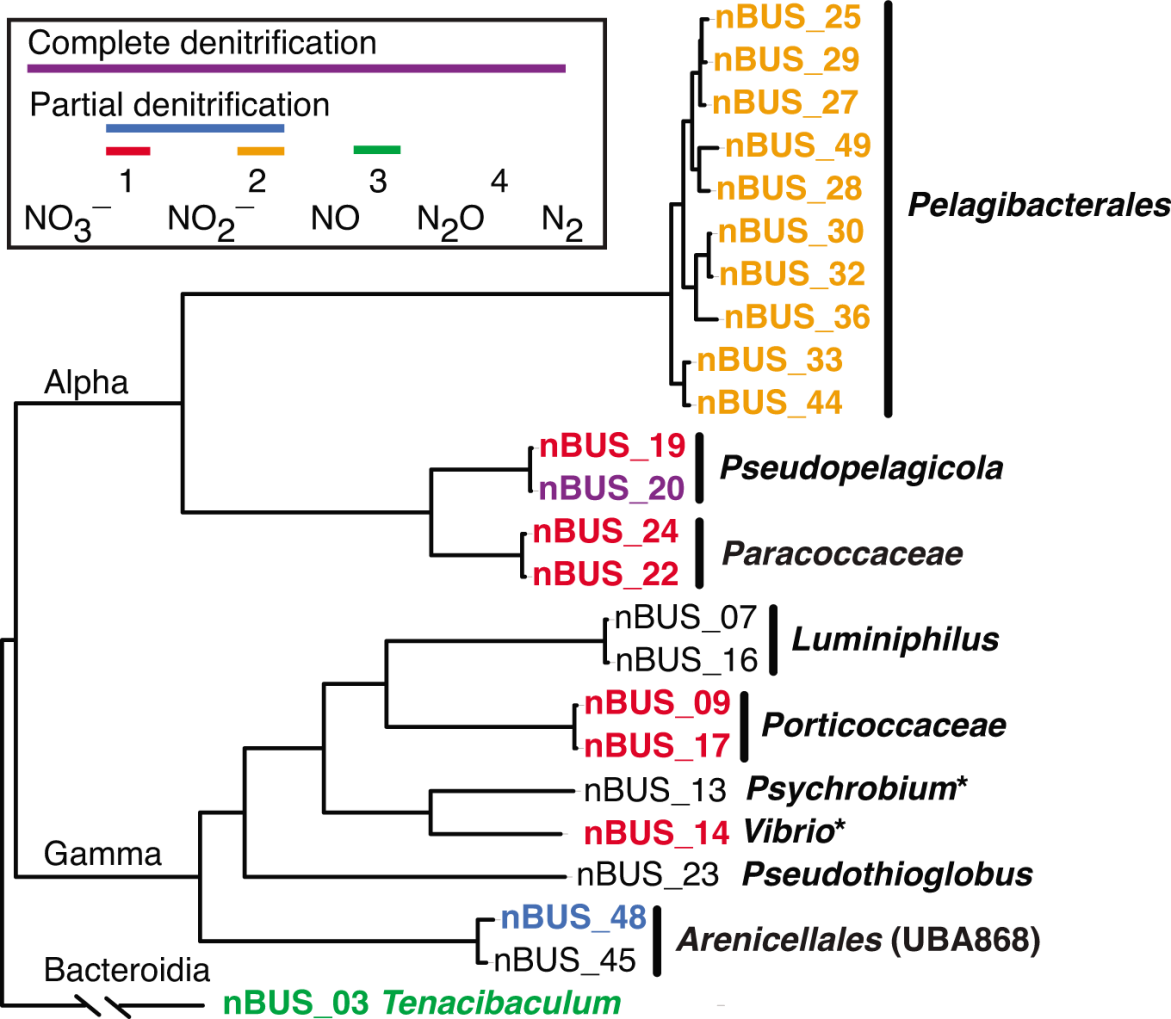
Whole-genome phylogeny of bacteria isolated and sequenced from the nBUS. Colours in the legend represent steps in denitrification (red, orange, green), or combinations of steps (blue, purple). Isolate Identification numbers (IDs) are colour-coded by the denitrification genes found in their genomes and correspond to the steps noted in the legend: nitrate reductases (*napAB* and *narGHI*), nitrite reductase (*nirK*), nitric oxide reductase (*norC*) and nitrous oxide reductase (*nosZ*). Isolates with no genes for denitrification are shown in black. Asterisks indicate the presence of assimilatory nitrogen reductases, *nasC* and *nirBD* genes, which are not considered canonical denitrification genes. KEGG orthology numbers associated with each isolate are in Tables S2 and S4.

Of the 51 marine bacteria isolated from the nBUS, 24 met our criteria for sequencing (see Methods). They include diverse members of the *Alphaproteobacteria*, *Gammaproteobacteria* and *Bacteroidia* (Fig. 1). Assembled genomes for all sequenced isolates are circular and range in size from 1.3 to 4.6 Mbp, with G+C contents between 29.6 and 52.5 mol% (Table S1, available in the online Supplementary Material). Most (19/24) have the genetic potential to carry out at least one step in the denitrification pathway. Only one isolate, *Pseudopelagicola* nBUS_20, has a full set of genes for complete denitrification, suggesting that it has the potential to reduce nitrate to N_2_ (Fig. 1, Table S2). Specifically, nBUS_20 codes for both periplasmic *napAB* and respiratory *narGHI* dissimilatory nitrate reductase genes, as well as a *nirS* nitrite reductase, *norC* nitric oxide reductase (NOR) and *nosZ* nitrous oxide reductase.

The potential to respire nitrate was broadly distributed among *Alphaproteobacteria* and *Gammaproteobacteria* (Fig. 1). The *Alphaproteobacteria* include *Pseudopelagicola* nBUS_19 and two representatives from a previously uncultured genus in the family *Paracoccaceae*, nBUS_22 and nBUS_24. The *Gammaproteobacteria* include two representatives from an undescribed genus in the family *Porticoccaceae* [32], nBUS_09 and nBUS_17, and *Vibrio* nBUS_14. One isolate (nBUS_48) encodes *narGHI* and *nirK* dissimilatory nitrate and nitrite reductase genes, indicating the potential for both dissimilatory nitrate and nitrite reduction. Isolate nBUS_48 is from a previously uncultured family of mixotrophs in the order *Arenicellales* (UBA868) that have the genetic potential to oxidize reduced sulphur throughout the mesopelagic zone [33].

Two isolates were identified with genes for assimilatory nitrate reduction. *Vibrio* nBUS_14 and *Psychrobium* nBUS_13 encode cytoplasmic *nasC* and *nirBD* assimilatory nitrate and nitrite reductases, respectively. The *nirBD* genes are generally associated with assimilatory nitrite reduction [34] but also have roles in fermentative dissimilatory nitrate reduction to ammonium (DNRA) [35]. Other key components of assimilatory nitrate reduction (*nasB*) and fermentative DNRA (*narGHI*) were not identified in these genomes, suggesting that the combination of cytoplasmic systems found in these isolates may represent an alternative pathway for nitrate reduction. The genome of the only *Bacteroidia* isolate, *Tenacibaculum* nBUS_03, codes for a single denitrification gene, *norC* NOR.

### Diversity of nBUS SAR11 isolates

**Fig. 2.**
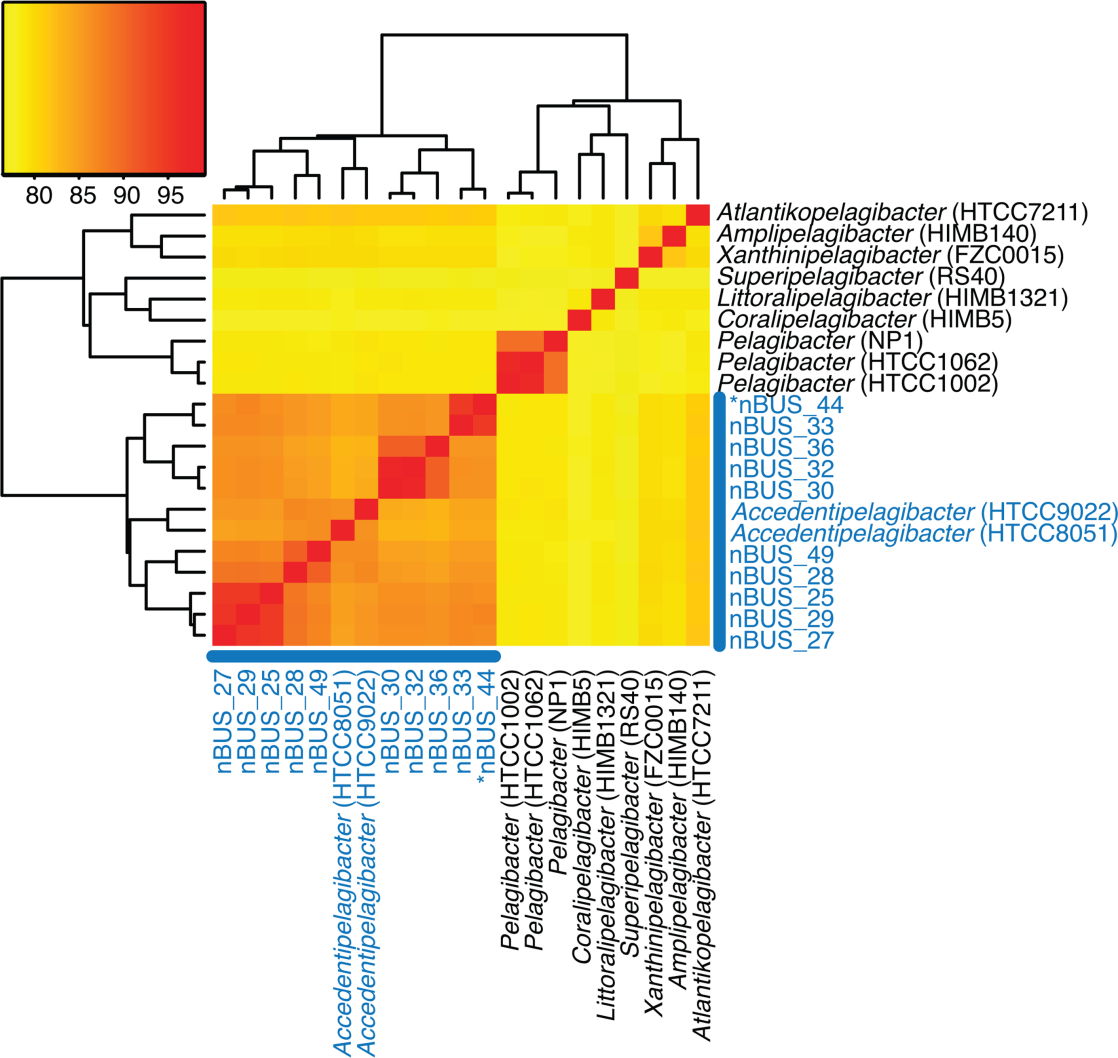
Pairwise ANI analysis of nBUS SAR11 genomes and those of previously cultured and sequenced genera. Labels correspond to genera designations [36] and nBUS isolate identification numbers (IDs). Blue text indicates *Proprepelagibacter* genus, and black text indicates other *Pelagibacter* genera. Asterisk indicates that the genome contains a prophage.

The ten SAR11 isolated from the nBUS are closely related species and subspecies, with genomes that range in size from 1.3 to 1.4 Mbp and have an average G+C content of 30.1 (±0.4) (Fig. 2, Table S1). Phylogenomic and whole-genome ANI analyses indicate that they represent six new species (ANI <95) and four new subspecies (ANI >95), all with pairwise ANI values >0.85 to subclade 1 a.III.IV (Table S3). This subclade was recently given the genus name *Accedentipelagibacter* [36].

### A *nirK*–cbb3 genomic region in nBUS SAR11

**Fig. 3.**
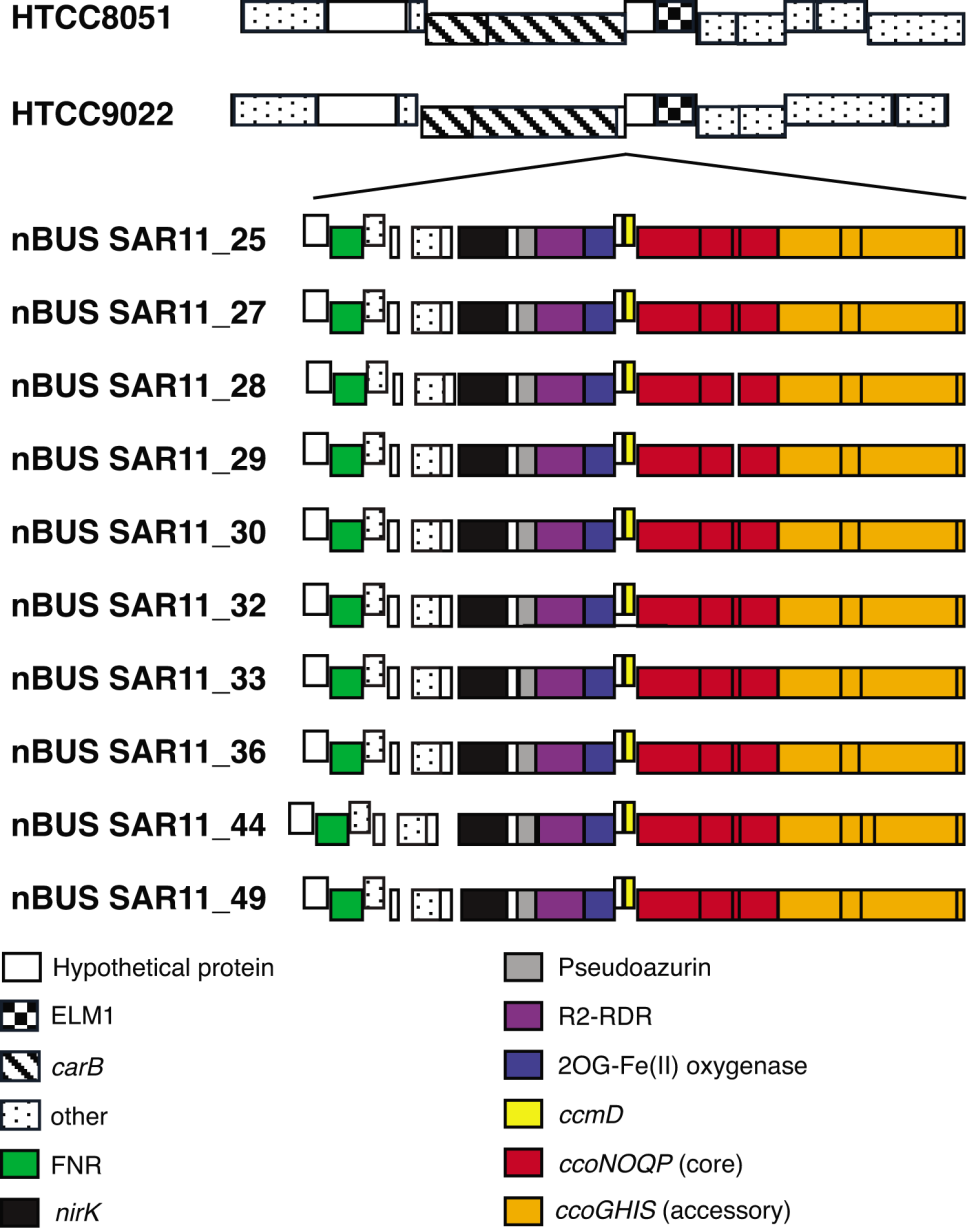
Genomic region with copper-containing *nirK* nitrite reductase and *cbb3*-type *cco* genes in nBUS SAR11 genomes. IDs are as follows: ELM1, mitochondrial fusion gene; *carB*, carbomyl-phosphate synthase; *crp-fnr*, transcriptional regulator; *nirK*, copper-containing nitrite reductase; R2-RDR, subunit beta ribonucleotide reductase; *ccm*D, cytochrome c biogenesis; *ccoNOQ* and *ccoPGHIS*, high-affinity *cco*. The genomic region insertion site is between the ELM1 and *carB* genes, indicated by lines pointing to the region in HTCC8051 and HTCC9022. Genes correspond to KEGG orthology numbers in Table S2.

The most striking feature in all ten nBUS SAR11 genomes is a conserved region with a copper-containing nitrite reductase (*nirK*) and components of cbb3-type cytochrome c oxidases (*cco*) that are not present in the complete genomes of cultured SAR11 from the North Pacific, North Atlantic and Indian oceans (Fig. 3, Table S4). The *nirK–cbb3* region is preceded by a *crp-fnr* transcriptional regulator. The FNR transcriptional regulator is required for the expression of anaerobic respiratory genes in *Escherichia coli* [37], suggesting that the *nirK–cbb3* genomic region in SAR11 may be expressed under low DO and anoxic conditions.

Similar *nirK* gene sequences were identified in seven MAGs obtained from Saanich Inlet [38] and in one SAR11 MAG obtained from an aquarium medusozoan cnidarian metagenome, *Cotylorhiza tuberculata* (OZ253498.1). Five of the Saanich Inlet MAGs failed Genome Taxonomy Database (GTDB) quality checks and had undefined taxonomies. Two were classified in GTDB as members of the HIMB59 clade of *Alphaproteobacteria* (GCA_018649225.1, GCA_018657885). Saanich Inlet MAGs also contained Fe(II)/2-oxoglutarate oxygenase and *crp-fnr* transcriptional regulator genes near the *nirK* gene. The *C. tuberculata* MAG belongs to the recently named *Amplipelagibacter* genus (clade Ib.4) with an ANI value of 92.8 to HIMB140. This MAG also contains pseudoazurin, Fe(II)/2-oxoglutarate oxygenase, key subunits of bd-type terminal oxidases and a *crp-fnr* transcriptional regulator gene near the *nirK* gene. These data suggest that diverse members of the SAR11 clade I and HIMB49 have the potential to reduce nitrite beyond the nBUS.

**Fig. 4.**
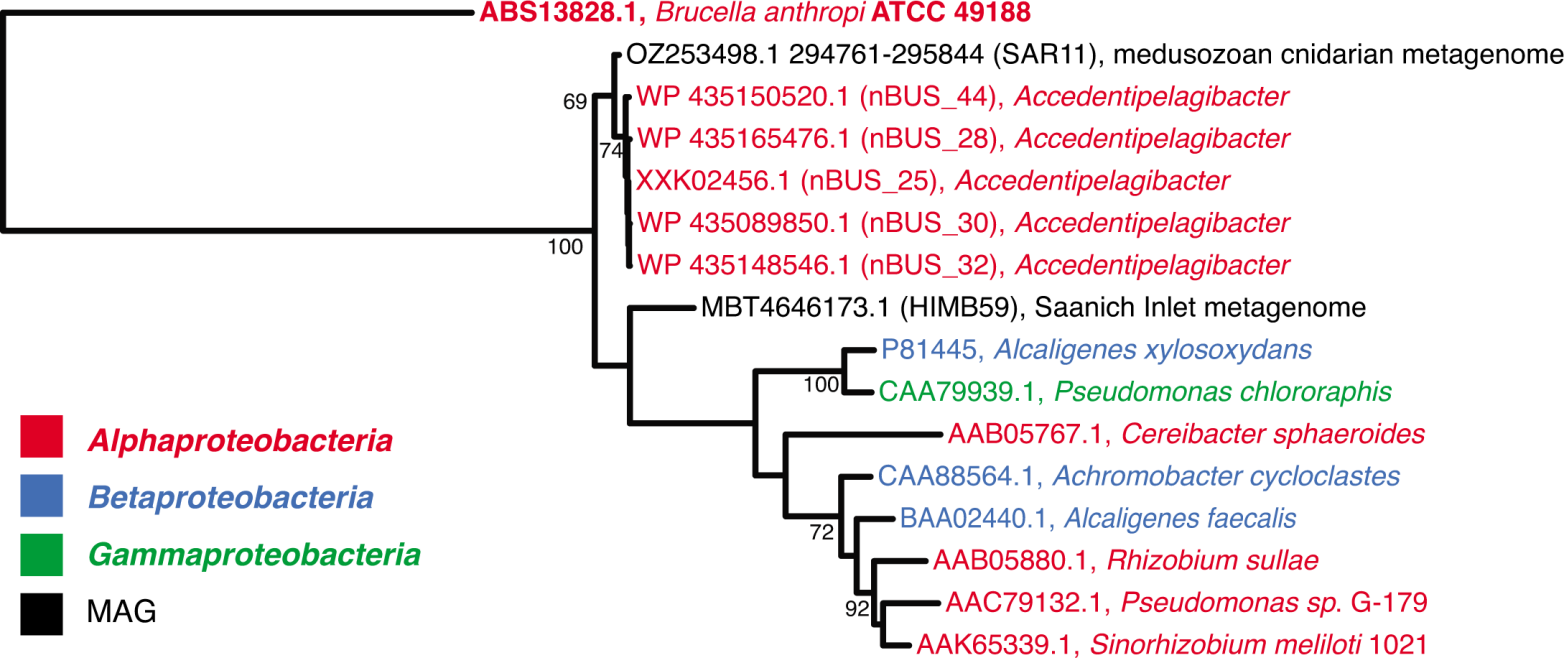
Comparison of NirK protein sequences from nBUS SAR11, a MAG from SI, a MAG from a medusozoan cnidarian (*C. tuberculata*) and several well-characterized denitrifying bacteria. A maximum-likelihood phylogenetic tree was constructed using type I NirK amino acid sequences. A type II NirK sequence was used as an outgroup (ATCC49188). SI, Saanich Inlet.

We then analysed the NirK proteins to evaluate phylogenetic relationships with known nitrite reductases and to look for known copper-binding sites and key active-site residues (Figs 4 and S1). We found that *nirK* in the SAR11 MAG from *C. tuberculata* was the most closely related sequence to NirK from nBUS SAR11 (Fig. 4). The amino acid sequence alignment verified the presence of known residues involved in copper binding and nitrite reduction [39] (Fig. S1), suggesting that the *nirK* gene in SAR11 has the potential to encode an active protein. Notably, putative SAR11 MAGs from Saanich Inlet also have protein-coding genes for nitrate transport and respiratory nitrate reduction, suggesting that some SAR11 may respire both nitrate and nitrite.

Genes for the nBUS cbb3-type *cco* are arranged in a canonical operon [40] that includes the catalytic subunit (*ccoN*), the cytochrome subunits involved in electron transfer (*ccoO* and *ccoP*), and a small subunit with an unknown function (*ccoQ*). This region also has accessory genes *ccoGHIS* [41]. The four genes between *nirK* and *ccoN* code for a protein of unknown function, a pseudoazurin, a Fe(II)/2-oxoglutarate oxygenase and subunit beta of ribonucleotide reductase (R2 RNR). Of these, only pseudoazurins have known roles contributing to denitrification [42,43].

None of the nBUS SAR11 genomes code for a NOR. However, Cco proteins share significant homology with NORs, which has led to considerable debate over the evolutionary origin of cbb3-type *cco*, especially regarding their roles in the evolution of aerobic respiration [40,44]. This also raises the possibility that some extant cbb3-type *cco* could be bifunctional enzymes that reduce oxygen under oxic conditions and NO to N_2_O under anoxic conditions. It has been known for some time that cbb3 haem–copper oxidases can reduce NO to N_2_O *in vivo* [45]. Interestingly, two of the genes we identified between *nirK* and the cbb3-type *cco* are related to proteins that bind NO, including the Fe(II)/2-oxoglutarate clavamate synthase from *Streptomyces clavuligerus* [46] and the R2 protein of ribonucleotide reductase from *E. coli* [47]. The R2 protein in *E. coli* not only binds NO but also produces N_2_O *in vivo*. Regardless, the presence of these genes suggests that SAR11 has the genetic potential for microaerophilic and anoxic growth and may contribute to denitrification in the nBUS.

## Conclusion

Genomic evidence obtained in this study suggests that denitrification potential in the nBUS is distributed among a diverse community of ubiquitous and abundant lineages of marine bacteria, including SAR11 (Fig. 1). Most of the bacteria we isolated and sequenced have the genetic potential to contribute to the initial steps in denitrification, either nitrate or nitrite reduction. Notably, all organisms with genes to respire nitrate or nitrite also have genes for cbb3-type *cco*, indicating the potential to respire oxygen at low concentrations. Relatively few have the genetic potential to contribute to more than one step in denitrification, and only one isolate was identified with the genetic potential for complete denitrification (*Pseudopelagicola* nBUS_20). Evidence that SAR11 have a genomic region with genes to reduce nitrite to NO suggests that they may represent an additional link in denitrification in the nBUS and elsewhere.

## Supplementary material

10.1099/mgen.0.001620 Uncited Fig. S1.

10.1099/mgen.0.001620 Uncited Supplementary Material 1.

## Abbreviations

anammox: anaerobic ammonia oxidation
ANI: average nucleotide identity
BUS: Benguela Upwelling System
*cco*: cytochrome c oxidases
DNRA: dissimilatory nitrate reduction to ammonium
DO: dissolved oxygen
ETNP: Eastern Tropical North Pacific
MAGs: metagenome-assembled genomes
nBUS: northern Benguela Upwelling System
NOR: nitric oxide reductase
OMZs: oxygen minimum zones
R2 RNR: subunit beta of ribonucleotide reductase
