# Supplementary material for: A nirK–cbb3 genomic region links SAR11 to nitrogen loss in the northern Benguela Upwelling System
Source: Microb Genom. 2026 Jan 19;12(1):001620. doi: 10.1099/mgen.0.001620 (PMC12816887; doi:10.1099/mgen.0.001620)

Supplementary Figure S1. Section of NirK amino acid sequence alignment with known residue involved in copper binding and nitrite reduction. Copper binding motifs T1Cu and T2Cu are highlighted in green and blue, respectively. Active site residues Asp and His, which are required for nitrite reducing activity, highlighted in yellow (39).

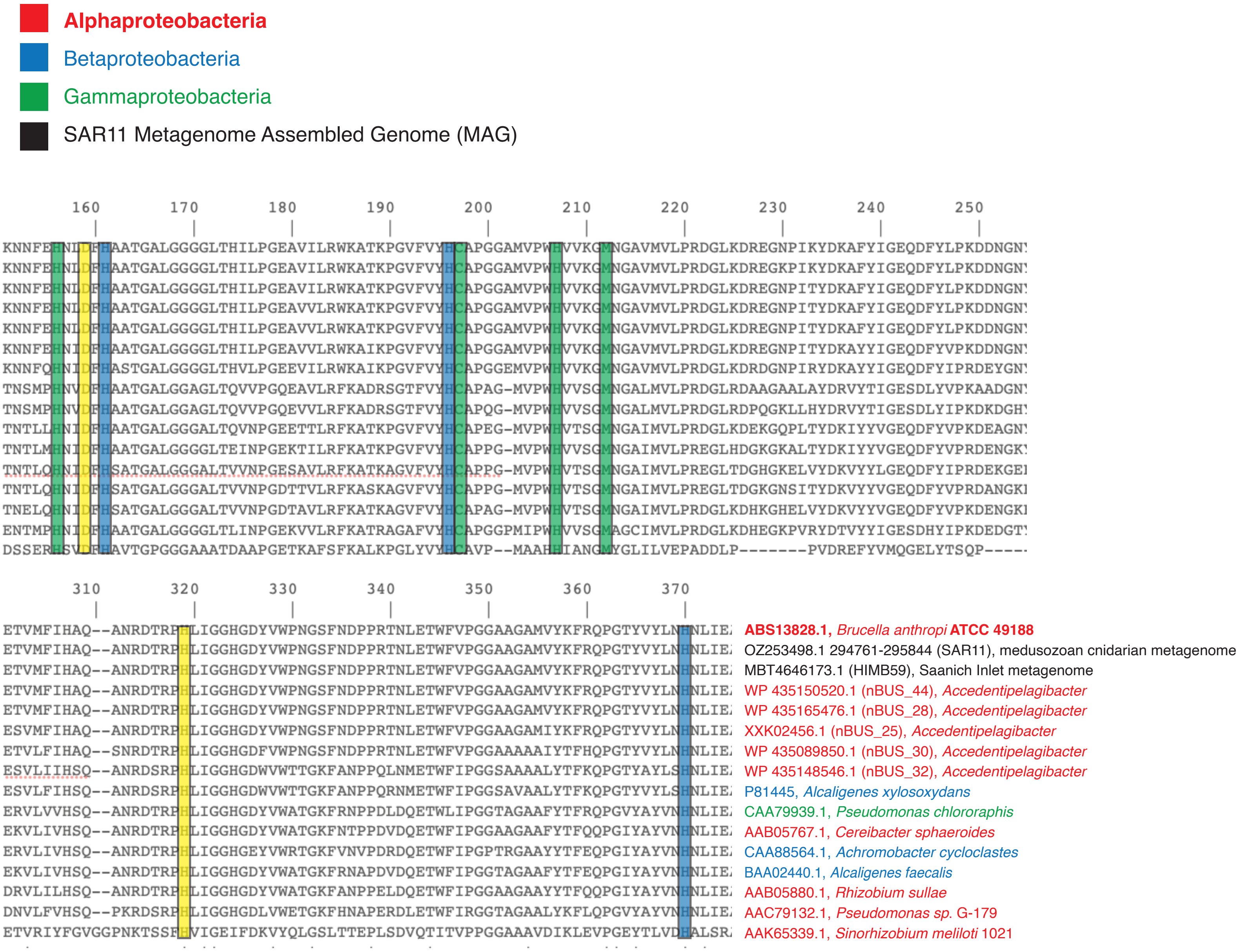

Supplement: Uncited Fig. S1. [file mgen-12-01620-s001.pdf]
